# Association of quality of life, anxiety, and depression with restless leg syndrome in the hemodialysis patients

**DOI:** 10.1186/s13104-021-05701-w

**Published:** 2021-07-23

**Authors:** Mahjabeen Yaseen, Furqan Ahmad Jarullah, Sadia Yaqoob, Hassan Abdullah Shakeel, Hamza Maqsood, Sadiq Naveed

**Affiliations:** 1 Fazaia Ruth Pfau Medical College, Karachi, Pakistan; 2 Jinnah Medical and Dental College, Karachi, Pakistan; 3 Nishtar Medical Univeristy, Multan, Pakistan; 4 Institute of Living, Hartford, CT USA

**Keywords:** RLS, Anxiety, Depression, Hemodialysis, Restless leg syndrome, Psychiatry

## Abstract

**Objectives:**

Restless Legs Syndrome (RLS) is commonly known to cause morbidity in patients on hemodialysis, making them prone to chronic mental health illnesses such as depression and anxiety, and also adversely impact quality of life. In this study, we examined the association of quality of life, anxiety, and depression with restless leg syndrome in the hemodialysis patients at Karachi Institute of Kidney Diseases.

**Results:**

About 26.7% of the participants reported RLS among the sample size Presence of RLS was not associated with quality of life, depression, and anxiety. However, p-values < 0.05 were significant for body-mass index (BMI), diabetes mellitus as a cause of end-stage renal disease, and serum albumin levels. Majority (82.5%) of the RLS-diagnosed patients had moderate to severe symptoms with 16 (40%) and 17 (42.5%) clients, respectively.

**Supplementary Information:**

The online version contains supplementary material available at 10.1186/s13104-021-05701-w.

## Introduction

Restless legs syndrome (RLS), also called Willis-Ekbom disease, is a common sensorimotor and neurological disorder that affects legs more than arms [1]. It is a clinical diagnosis. A standard criterion was published by the International Restless Legs Syndrome Study Group (IRLSSG) in 1995 and later revised in 2002 [2, 3]. RLS symptoms include an unpleasant or creeping sensation and an irresistible desire to move the limbs. The symptoms usually worsen at night and at times of physical inactivity and are partly relieved by movement [4].

RLS's estimated prevalence rate in hemodialysis patients is 6.6–70% [5], while in the general population; its prevalence is only 3–9% [6]. RLS is classified into two categories- primary and secondary based on underlying etiology. Approximately 40% of the primary RLS cases are idiopathic with a strong familial predisposition [2], whereas secondary RLS is usually associated with iron deficiency anemia, pregnancy, diabetes, uremia, peripheral neuropathy, rheumatoid arthritis, and certain medications, namely anti-depressants and anti-hypertensives [1, 7]. The risk factors include iron deficiency anemia, abnormal calcium, phosphorus and parathyroid hormone levels, duration of hemodialysis, increased body mass index (BMI), and female gender.

Existing literature have explored the evidence for frequent occurrence of psychiatric comorbidity such as depression, anxiety, and insomnia in patients with RLS [8]. RLS has been considered as a risk factor for depression but recent studies also suggests a bidirectional relationship between RLS and depression [9]. This can be attributed to the chronic course of RLS, needing long-term treatment, and impact on the quality of life. The evidence is scarce in the context of low and middle income countries such as Pakistan. In this cross-sectional study, we intended to provide evidence pertaining to Pakistan regarding RLS's prevalence in hemodialysis patients and its association with life quality, depression and anxiety. With fewer studies concentrating on finding an association of RLS with anxiety and depression, our motive is to find a correlation.

## Main text

### Methods

This observational, cross-sectional, and single-centered study was carried out at the Karachi Institute of Kidney Diseases (KIKD). The data collection period lasted from July to December 2019.Informed consent from the participants was obtained before carrying out a face-to-face interview. We included all the patients above 18 years of age who were on hemodialysis for more than three months in our study. Any participant who had Parkinson's disease, psychosis, arthritis, and muscle pain was excluded from the study. Besides that, patients with incomplete labs and those who refused to provide consent were also excluded.

The questionnaire has four components. The first part comprises the socio-demographic and biochemical parameters of patients; the second one comprised of International Restless Legs Syndrome Study Group (IRLSSG) diagnosis and severity scales; the third section included the World Health Organization Quality of Life (WHO-QOL)-BREF scale and, the fourth part included the Hospital Anxiety and Depression Scale (HADS) scale. The socio-demographic and biochemical parameters encompassed age, gender, BMI, cause of renal failure, duration of hemodialysis, number of dialysis per week, a shift of dialysis, Hemoglobin levels, pre-dialysis urea, post-dialysis urea, calcium, phosphorous, albumin, and parathyroid hormone levels.

We used IRLSSG 2003 diagnostic criteria to diagnose RLS in participants [10]. The following four essential criteria must be fulfilled to diagnose RLS:

- (1) An urge to move the legs, usually but not always accompanied by or felt to be caused by uncomfortable and unpleasant sensations in the legs.
- (2) The urge to move the legs and any accompanying unpleasant sensations begin or worsen during rest or inactivity such as lying down or sitting.
- (3) The urge to move the legs and any accompanying unpleasant sensations are partially or relieved by movement, such as walking or stretching, at least as long as the activity continues.
- (4) The urge to move the legs and any accompanying unpleasant sensations during rest or inactivity only occur or are worse in the evening or night than during the day.

Following the diagnosis of RLS, IRLSSG Severity Scale was used to assess the severity of RLS symptoms. Those with a score of 31–40 were categorized as very severe; a score of 21–30, as severe; 11–20, as moderate, and 0–10, as mild.

The World Health Organization Quality of Life (WHO-QOL)-BREF questionnaire was used to assess the quality of life in hemodialysis patients [11]. It is a self-reporting questionnaire comprising 26 questions, which gauges the quality of life across four facets; physical, social, psychological, and environmental. Sensitivity for overall quality of life and overall health was evaluated independently. Responses to questions are on a 1–5 Likert scale where one denotes 'disagree' or 'not at all' and 5 denotes 'completely agree' or extremely. The raw score obtained from the WHOQOL-BREF was transformed into a linear scale between 0 and 100 following the scoring guidelines [12, 13]. A higher score indicated a better QOL.

The Hospital Anxiety and Depression Scale (HADS) was used to screen patients for anxiety and depression [14]. HADS is a fourteen-item scale with seven items in each subscale; anxiety, and depression. A score of 0–7 is regarded as normal, 8–10 as borderline, and 11–21 as an abnormal case for each subscale.

We used SPSS version 25.0 (IBM Corp, New York USA) for the data analysis. Categorical variables were reported as frequencies and percentages and continuous variables as means and standard deviations. An independent two-sample t-test was run to compare the means of continuous variables between the hemodialysis patients with and without RLS, and the Chi-squared test was used to observe the association between the categorical variables of the two study groups. Median scores on the WHOQOL-BREF domains of RLS positive and negative patients were obtained using the Mann–Whitney U test. Results were interpreted with 95% confidence intervals, and a p-value of less than 0.05 was considered significant.

### Results

One hundred fifty participants were eligible for the final investigation of which, 56% (84 patients) were males, and the remaining 44% (66 patients) were females. The mean age of the participants was 47.82 ± 13.53 years. Out of 40 RLS-positive patients, 29 (72.5%) were males, and this finding was significant with a p-value of 0.014. Of the total RLS-negative participants, there was an equal 1: 1 ratio of both genders. The average BMI of the sample was 24.09 ± 5.48. However, there was a clear difference between the Body Mass Indices of the two groups of positive and negative RLS patients. As seen in Table 1, the p-value was 0.005 for this modality. 105 (70%) participants claimed that the cause of renal failure was hypertension, followed by diabetes mellitus (38%).

**Table 1:** Socio-demographic and clinical parameters for RLS positive and negative patients undergoing hemodialysis

Patient’ characteristics	Total sample (N = 150)	RLS positive (n = 40)	RLS negative (n = 110)	p-value
Age, years (mean ± SD)	47.82 ± 13.53	13.57 ± 2.15	13.55 ± 1.29	0.980
Gender (%)				
Male	84(56.0)	29(72.5)	55(50.0)	0.014*
Female	66(44.0)	11(27.5)	55(50.0)	
BMI, kg/m^2^ (mean ± SD)	24.09 ± 5.48	23.86 ± 4.55	24.18 ± 5.80	0.005*
Duration of Dialysis, years (mean ± SD)	2.35 ± 2.63	2.44 ± 0.39	2.70 ± 0.26	0.475
Dialysis/week (mean ± SD)	2.49 ± 0.56	0.55 ± 0.09	0.57 ± 0.05	0.665
Shift (%)				
Morning	55(36.7)	14(35.0)	41(37.3)	0.678
Afternoon	45(30.0)	14(35.0)	31(28.2)	
Evening	50(33.3)	12(30.0)	38(34.5)	
Causes of ESRD (%)				
Diabetes Mellitus	57(38.0)	21(52.5)	36(32.7)	0.027*
Hypertension	105(70.0)	29(72.5)	76(69.1)	0.687
Glomerulonephritis	6(4.0)	2(5.0)	4(3.6)	0.706
Renal stones	13(8.7)	3(7.5)	10(9.1)	0.759
Others	20(13.3%)	3(7.5)	17(15.5)	0.205
Patient clinical characteristics (mean ± SD)				
Pre dialysis urea (mmol/l)	106.77 ± 58.61	109.95 ± 48.03	105.61 ± 62.17	0.603
Post dialysis urea (mmol/l)	101.49 ± 101.90	86.99 ± 32.31	106.76 ± 117.11	0.149
Hb (g/l)	10.36 ± 2.01	10.74 ± 2.74	10.22 ± 1.80	0.165
Calcium (mmol/l)	8.39 ± 2.00	8.20 ± 2.18	8.46 ± 1.93	0.336
Phosphorous (mmol/l)	5.41 ± 2.03	5.31 ± 1.98	5.45 ± 2.06	0.981
Albumin (g/l)	4.64 ± 3.77	5.46 ± 5.81	4.34 ± 2.64	0.004*
Parathyroid hormone (mmol/l)	380.87 ± 366.83	418.68 ± 425.04	367.12 ± 344.38	0.061

^*^Significant p-value (p < 0.05)

The estimated prevalence of RLS was 26.7%. It was also noted that between the groups of RLS-positive and RLS-negative clients, there was a significant difference between those who were formerly diagnosed with Diabetes Mellitus (DM). Twenty one (52.5%) subjects with RLS had concurrent DM, with a significant p-value of 0.027. Analysis of laboratory results revealed a substantial difference between albumin levels in the two groups, which was remarkably higher in RLS-positive patients. These results are shown in Table 1.

**Fig. 1:**
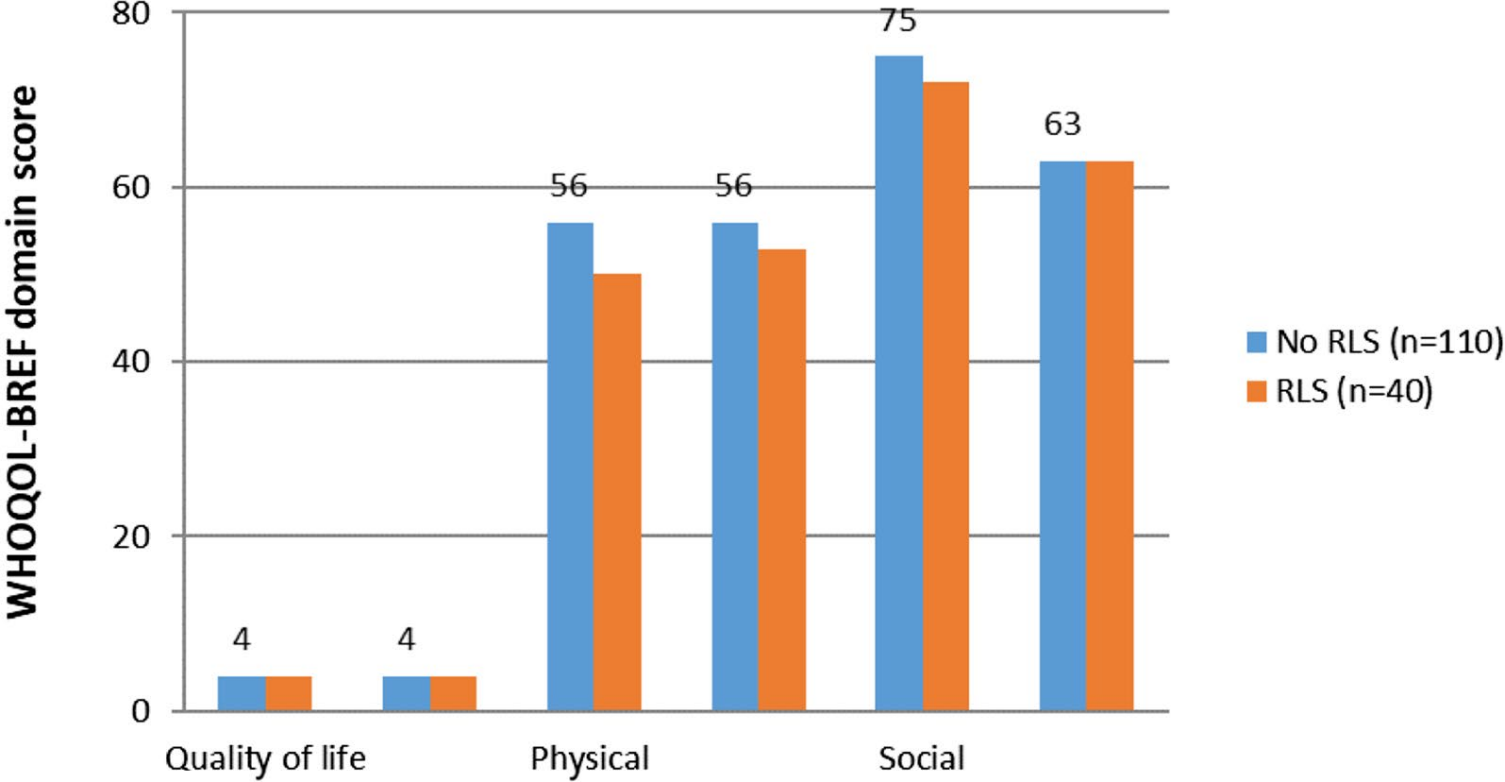
Median scores on the WHOQOL-BREF domains of RLS positive and negative patients. Differences between the two groups were tested with the Mann–Whitney U test. P-values were not significant (p > 0.05) for all domains

Mann–Whitney U test was also performed to see any statistical significance in the WHO-QOLBREF scale scores. Results displayed in Fig. 1 reveal that the scores across all the domains- general health, quality of life, physical, psychological, social, and environmental had p-values > 0.05 and hence, statistically insignificant.

As observed in Table 2, the majority (82.5%) of the RLS-diagnosed patients had moderate to severe symptoms with 16 (40%) and 17 (42.5%) clients, respectively. Six (15%) of the remaining participants had only mild severity, and only 1 (2.5%) patients had very severe RLS symptoms.

**Table 2:** Severity of RLS according to IRLSSG rating scale

Severity of RLS	Frequency (N = 40)	Percentage (%)	Average score (MEAN ± SD)
Mild	6	15	9.33 ± 1.03
Moderate	16	40	16.06 ± 2.24
Severe	17	42.5	24.53 ± 2.55
Very severe	1	2.5	–
Total score	40	100	19.08 ± 6.40

A chi-square test was applied to see if there was a significant difference between RLS-morbid and non-morbid groups regarding anxiety and depression measured via the HADS scale. This test generated inconsequential results as the values of significance for depression and anxiety were 0.875 and 0.085, respectively, as illustrated in Additional file 1: Table S1.

### Discussion

The prevalence of RLS in our study was 26.7%, which is substantially higher compared to a number of studies reporting prevalence from 15.8–20.44% [1, 4, 7, 15]. However, it is lower compared to the prevalence reported by Al-Jahdali et al. (50.22%) [16] and Giannaki et al. (27.1%) [17].

In a systematic review, the reported prevalence was 34% among the general population undergoing hemodialysis. The prevalence is 17.7% for the participants under peritoneal dialysis [18]. This higher prevalence is likely due to decrease renal function that is a risk factor for RLS [18]. The varying prevalence is also reported due to ethnic variations such as Greeks and Indians have been reported to have a lower prevalence of RLS [19, 20]. Although, there is no clear mechanism defining this association, it has been attributed to anemia, deficiency of iron and vitamins [18].

Our study failed to find an association between low albumin, high urea, and low hemoglobin with RLS. These results are not consistent with the finding of Turk et al. reporting significant association between low albumin levels with RLS, as suggested by a p-value < 0.05 [1]. Similarly, Lin et al. reported that urea levels were higher in RLS-positive patients [15]. Furthermore, Giannaki et al. reported lower hemoglobin levels in RLS patients, which were not analogous to our results [17]. However, no significant difference in urea, albumin and hemoglobin was observed in RLS positive individuals (n = 25) and RLS negative individuals (n = 93) enrolled in a study conducted by Salman et al. [21]. Our findings are preliminary and warrant further replicate studies to help resolve the controversies and measure these laboratory indices.

Beladi-Mousavi et al. suggested that most RLS diagnosed patients experienced severe symptoms (40.9%), comparable to 42.5% estimated in our study population. However, only 2.5% were subjected to the very severe discomfort category in our research, unlike 9.09% reported by Beladi-Mousavi et al. [7]. Giannaki et al. also implied that general health was a factor that was notably decreased in RLS-positive patients [17]. Kutlu R et al. tabulated that physical, social, and environmental parameters showed their repercussions in RLS-positive individuals [22]. Mucsi I et al. stated that all domains of the quality of life were affected except for the physical domain [23]. Our study, in contrast to the studies above, does not show similar results that may indicate any impairment of QOL amongst RLS patients on hemodialysis. Even though Yilmaz et al. ascertained that depression and anxiety co-existed in RLS [24], we failed to establish any significant correlation. Such a variation in data may arise from socio-cultural factors affecting the individual’ mood and causing remission and relapse in anxiety and depression [25].

### Conclusion

RLS is frequently reported in hemodialysis patients and should be evaluated and treated early to reduce morbidity. Even though these patients' prevalence was remarkably high, we failed to establish any association of RLS with quality of life, anxiety, and depression. Further studies are required to confirm whether these predictors contribute to the disease in any manner.

## Limitations

Self-reporting nature of the questionnaires may introduce recall bias in the study. Moreover, due to sensitive nature of the information, respondents might have underreported information about quality of life. Due to its cross-sectional design, causal relationships cannot be established between two variables. Despite a higher sample size and an inclusive study sample, the results of this investigation should not be generalized to the whole Pakistani population. In addition, association of quality of life, anxiety and depression in restless syndrome patients undergoing hemodialysis were evaluated using dichotomous questions, which may not be a sensitive measure of these constructs.

## Supplementary Information

**Additional file 1: Table S1.** Chi-square tests comparing patients with and without RLS based on depression (HADS-D) and anxiety (HADS-A).

## Data Availability

A confidentiality agreement with participants prevents us from sharing the data, therefore, dataset cannot be shared.

## Abbreviations

RLS: Restless leg syndrome
BMI: Body mass index
IRLSSG: International Restless Legs Syndrome Study Group
WHO-QOL: World Health Organization Quality of Life
HADS: Hospital anxiety and depression scale

## References

[1] Turk AC, Ozkurt S, Turgal E, Sahin F (2018). The association between the prevalence of restless leg syndrome, fatigue, and sleep quality in patients undergoing hemodialysis. Saudi Med J.

[2] Walters AS, LeBrocq C, Dhar A (2003). Validation of the International Restless Legs Syndrome Study Group rating scale for restless legs syndrome. Sleep Med.

[3] Buysse DJ, Reynolds CF, Monk TH, Berman SR, Kupfer DJ (1989). The Pittsburgh Sleep Quality Index: a new instrument for psychiatric practice and research. Psychiatry Res.

[4] Wali SO, Alkhouli AF (2015). Restless legs syndrome among Saudi end-stage renal disease patients on hemodialysis. Saudi Med J.

[5] Lin CH, Wu VC, Li WY (2013). Restless legs syndrome in end-stage renal disease: a multicenter study in Taiwan. Eur J Neurol.

[6] Trenkwalder C, Paulus W, Walters AS (2005). The restless legs syndrome. Lancet Neurol.

[7] Beladi-Mousavi SS, Jafarizade M, Shayanpour S, Bahadoram M, Moosavian SM, Houshmand G (2015). Restless Legs Syndrome: associated risk factors in hemodialysis patients. Nephrourol Mon..

[8] Winkelmann J, Prager M, Lieb R (2005). "Anxietas tibiarum". Depression and anxiety disorders in patients with restless legs syndrome. J Neurol.

[9] Lee HB, Hening WA, Allen RP (2008). Restless legs syndrome is associated with DSM-IV major depressive disorder and panic disorder in the community. J Neuropsychiatry Clin Neurosci.

[10] Allen RP, Picchietti D, Hening WA (2003). Restless legs syndrome: diagnostic criteria, special considerations, and epidemiology A report from the restless legs syndrome diagnosis and epidemiology workshop at the National Institutes of Health. Sleep Med.

[11] Feder K, Michaud DS, Keith SE, et al. An assessment of quality of life using the WHOQOL-BREF among participants living in the vicinity of wind turbines. Environmental Research. https://www.sciencedirect.com/science/article/pii/S0013935115300189. . Accessed 12 Nov 2020. 10.1016/j.envres.2015.06.043 26176420

[12] Skevington SM, Tucker C (1999). Designing response scales for cross-cultural use in health care: data from the development of the UK WHOQOL. Br J Med Psychol.

[13] Programme on mental health : WHOQOL user manual. World Health Organization. https://apps.who.int/iris/handle/10665/77932. Accessed 12 Nov 2020.

[14] Zigmond AS, Snaith RP (1983). The hospital anxiety and depression scale. Acta Psychiatr Scand.

[15] Lin XW, Zhang JF, Qiu MY (2019). Restless legs syndrome in end stage renal disease patients undergoing hemodialysis. BMC Neurol.

[16] Al-Jahdali HH, Al-Qadhi WA, Khogeer HA, Al-Hejaili FF, Al-Ghamdi SM, Al Sayyari AA (2009). Restless legs syndrome in patients on dialysis. Saudi J Kidney Dis Transpl.

[17] Giannaki CD, Hadjigavriel M, Lazarou A (2017). Restless legs syndrome is contributing to fatigue and low quality of life levels in hemodialysis patients. World J Nephrol.

[18] Ghanei Gheshlagh R, Farajzadeh M, Zarei M, Baghi V, Dalvand S, Sayehmiri K (2017). The prevalence of restless legs syndrome in patients undergoing hemodialysis: a systematic review and meta-analysis study. Basic Clin Neurosci.

[19] Hadjigeorgiou GM, Stefanidis I, Dardiotis E (2007). Low RLS prevalence and awareness in central Greece: an epidemiological survey. Eur J Neurol.

[20] Bhowmik D, Bhatia M, Gupta S, Agarwal SK, Tiwari SC, Dash SC (2003). Restless legs syndrome in hemodialysis patients in India: a case controlled study. Sleep Med.

[21] Salman SMY (2011). Restless legs syndrome in patients on hemodialysis. Saudi J Kidney Dis Transplant.

[22] Kutlu R, Selcuk NY, Sayin S, Kal O (2018). Restless legs syndrome and quality of life in chronic hemodialysis patients. Niger J Clin Pract.

[23] Mucsi I, Molnar MZ, Ambrus C (2005). Restless legs syndrome, insomnia and quality of life in patients on maintenance dialysis. Nephrol Dial Transplant.

[24] Yilmaz O, Şengül Y, Şengül HS, Parlakkaya FB, Öztürk A (2018). Investigation of alexithymia and levels of anxiety and depression among patients with restless legs syndrome. Neuropsychiatr Dis Treat.

[25] Kessler RC, Bromet EJ (2013). The epidemiology of depression across cultures. Annu Rev Public Health.

