# Supplementary material for: Association of quality of life, anxiety, and depression with restless leg syndrome in the hemodialysis patients
Source: BMC Res Notes. 2021 Jul 23;14:284. doi: 10.1186/s13104-021-05701-w (PMC8306279; doi:10.1186/s13104-021-05701-w)
Supplement: Supplementary file 1 — Additional file 1: Table S1. Chi-square tests comparing patients with and without RLS based on depression (HADS-D) and anxiety (HADS-A). [file 13104_2021_5701_MOESM1_ESM.doc]

| **Variables** | **RLS** | **No RLS** | **P-value** |
| --- | --- | --- | --- |
| **Depression** |  | | 0.875 |
| Normal  Borderline-Abnormal  Abnormal | 22 (55%) | 56 (50.9%) |  |
|  | 9 (22.5%) | 29 (26.4%) |  |
|  | 9 (22.5%) | 25 (22.7%) |  |
| **Anxiety** |  | | 0.085 |
| Normal  Borderline Abnormal  Abnormal | 27 (67.5%) | 54 (49.1%) |  |
|  | 10 (25%) | 34 (30.9%) |  |
|  | 3 (7.5%) | 22 (20%) |  |

**Table S1 :** Chi-square tests comparing patients with and without RLS based on depression (HADS-D) and anxiety (HADS-A).
